# Parent–child communication and adolescent problematic internet use: A cross-sectional dyadic study of protective effects and the primacy of adolescents' own perceptions

**DOI:** 10.1016/j.abrep.2026.100733

**Published:** 2026-08-07

**Authors:** Nobuaki Morita

**Affiliations:** Institute of Medicine, University of Tsukuba, Japan

**Keywords:** Parent–child communication, Problematic internet use, Discrepancy, Behavioral addiction, Family processes, Adolescence

## Abstract

**Background:**

Adolescent problematic internet use (PIU) has been linked to family processes, including intergenerational patterns. Although parent–child communication is often considered protective, whether it buffers intergenerational risk or functions independently remains unclear.

**Methods:**

In this cross-sectional dyadic study, 824 Japanese high school students (aged 15–20, M = 16.7, SD = 0.8) and their biological parents completed parallel online questionnaires assessing PIU and communication via a commercial research panel (Macromill). Hierarchical regressions examined direct, moderating, and discrepancy effects, controlling for parent demographics.

**Results:**

Parental PIU was positively associated with adolescent PIU (β = 0.53, *p* < .001). Higher mean-level communication—the average of parent and adolescent reports—was associated with lower adolescent PIU (parental acceptance: β = −0.08, *p* < .05; low perceived barriers to consultation: β = −0.13, *p* < .001), but did not moderate the parent–adolescent PIU association (both *p* > .20). Adolescents' own reports of low perceived barriers to consultation (β = −0.51) were far more strongly associated with adolescent PIU than parents' reports (β = 0.10, ns); response surface analysis indicated asymmetric informant weighting (a3 = 0.61, *p* < .001) rather than a non-linear discrepancy effect (a4 = −0.01, ns). Discrepancies in parental acceptance were unrelated to adolescent PIU. Findings were unchanged after controlling for parent sex and age.

**Conclusions:**

Parent–child communication is an independent protective factor rather than a buffering mechanism. Adolescents' own perceptions of help-seeking barriers, more than discrepancies per se, were associated with risk, meriting attention in future research pending longitudinal, multi-method replication.

## Highlights

• Parent-child communication predicted lower adolescent problematic internet use.
• Communication did not buffer the parent-adolescent PIU link, for either dimension.
• Adolescents' own reports of low barriers to consultation predicted their PIU.
• Parents' reports of barriers were largely unrelated to adolescent PIU.
• Discrepancies reflected asymmetric informant weighting, not a nonlinear pattern.

## 1 Introduction

Problematic Internet Use (PIU) is increasingly categorized as a behavioral addiction (Fineberg et al., 2018), though its conceptualization and measurement remain debated (Billieux et al., 2026; Fineberg et al., 2025); we treat PIU as a pattern of impaired control, functional impairment, and continued use despite negative consequences, while acknowledging that consensus on formal diagnostic criteria has not been reached (Fournier et al., 2023). In Japan and other Asian countries, PIU prevalence is estimated at 7.0–18.7%, a critical public health concern (Mihara et al., 2016; Pan et al., 2020). PIU among adolescents has serious implications for physical and mental development, with family relationships increasingly recognized as a contributing factor.

Emerging evidence suggests that PIU exhibits intergenerational patterns: adolescents of parents with PIU are substantially more likely to exhibit PIU themselves (Milkovich et al., 2025), a correspondence observed across diverse national contexts (Yankouskaya et al., 2025), highlighting the importance of family-level mechanisms**.**

Parent–child communication has been widely regarded as a protective factor, with higher parental acceptance, openness, and supportive communication generally associated with better psychological outcomes and lower risk behaviors (Steinberg, 2001).

Parent–child communication here refers to the perceived quality (not frequency) of communication about internet use, encompassing two conceptually distinct aspects: adolescents' general sense that parents are non-judgmental and understanding (parental acceptance), and their sense of being able to approach parents specifically about internet-related difficulties (low perceived barriers to consultation). This distinction mirrors an established contrast in the parenting literature between broad relational climate and situation-specific help-seeking accessibility (De Los Reyes & Kazdin, 2005; Steinberg, 2001). We focus on these two aspects because parallel parent- and adolescent-report versions of each were available, enabling the multi-informant analyses central to this study; the instrument used to operationalize these constructs, and its psychometric properties, are described in the Method section.

We distinguish two roles of communication: a protective effect (a direct association, or main effect, between higher communication and lower adolescent PIU) and a buffering effect (a moderating process, or interaction between parental PIU and communication, whereby communication weakens the parent–adolescent PIU link)—a distinction that matters because family research often assumes positive relational processes buffer risk when they may instead operate independently.

Whether communication buffers or instead operates independently of parental risk is not merely definitional. Modeling accounts of intergenerational transmission (Bandura, 1977) suggest that adolescents may internalize observed parental behavior regardless of relationship quality, and parental smartphone preoccupation (“phubbing”) has been shown to predict adolescent PIU irrespective of communication quality (Ma et al., 2024). Such evidence raises the empirical possibility that parental modeling and communication operate through separate pathways rather than one moderating the other, motivating a direct test of buffering rather than an assumption that it occurs.

A large parental mediation literature shows active parental involvement is associated with healthier adolescent media use (Livingstone et al., 2017; Sevilla-Fernández et al., 2025), though this research has focused on direct effects, with less attention to whether communication moderates intergenerational associations—an assumption implicit in much of the field but with inconsistent empirical support.

An alternative perspective is that communication functions as an independent protective factor without necessarily modifying intergenerational transmission.

This connects to the multi-informant literature in developmental psychopathology (De Los Reyes, 2013; De Los Reyes & Kazdin, 2005), which holds that low parent–adolescent agreement often reflects genuinely different vantage points rather than measurement error: a parent may believe they are approachable while their adolescent does not share that perception, a mismatch that could matter more for outcomes than either report alone, since it captures a breakdown in the adolescent's subjective experience of accessibility to help-seeking. The direction of any discrepancy may also carry distinct meaning, a question we return to later.

Despite these advances, two gaps remain: whether parent–child communication functions as a buffering mechanism or an independent protective factor, and whether discrepancies between parent and adolescent perceptions of communication play a role in adolescent PIU.

**Fig. 1:**
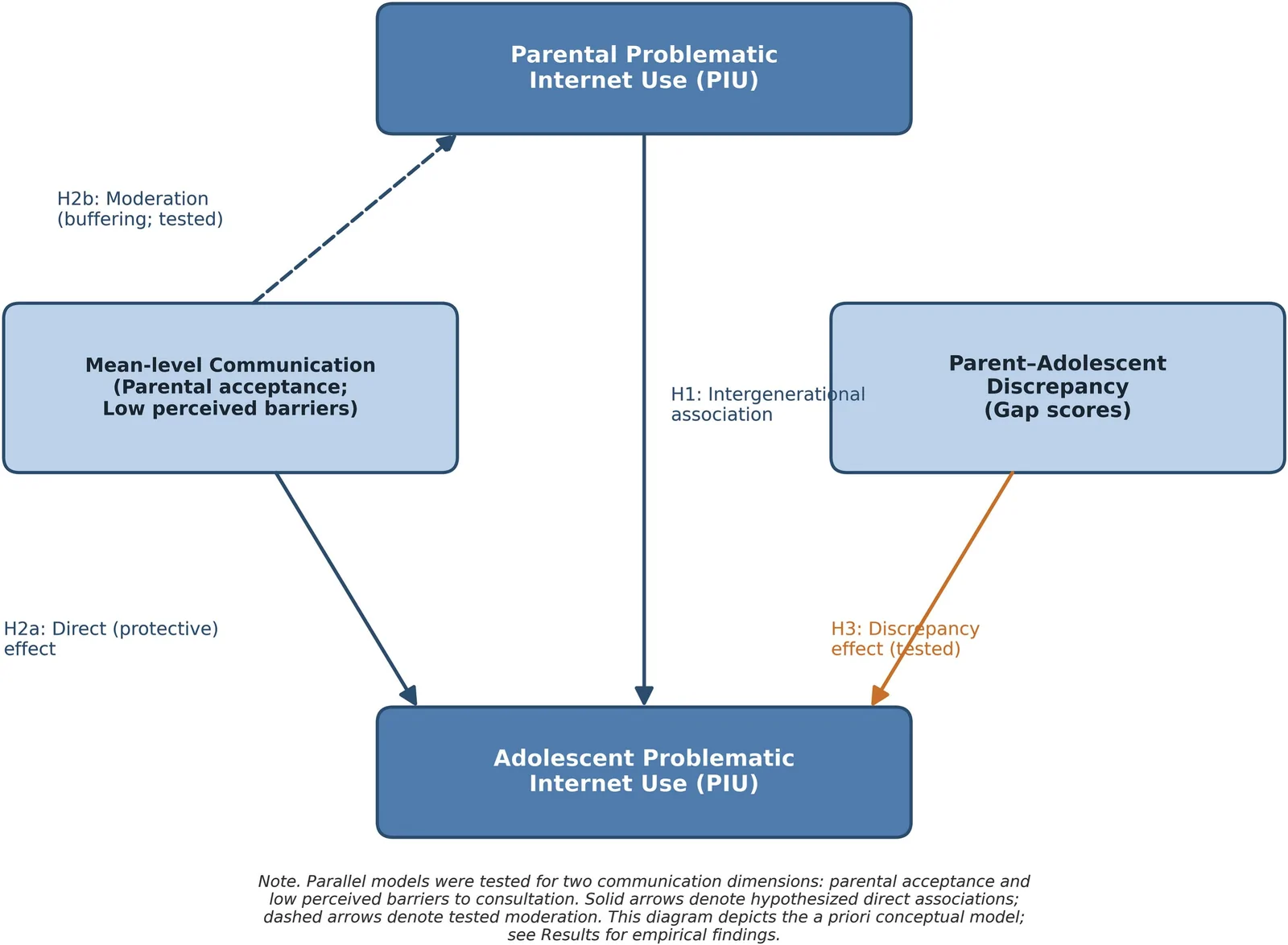
**Conceptual model of the study.** The a priori model depicts the hypothesized intergenerational association between parental and adolescent problematic internet use (H1), the hypothesized direct and moderating (buffering) roles of mean-level communication (H2a, H2b), and the hypothesized effect of parent–adolescent discrepancy (H3), tested in parallel for parental acceptance and low perceived barriers to consultation. See Results for empirical findings.

The present study addresses these gaps by examining, in parallel for parental acceptance and low perceived barriers to consultation: (1) whether higher mean-level communication—the average level of communication reported across the parent–adolescent dyad—is directly associated with lower adolescent PIU; (2) whether communication moderates (buffers) the parent–adolescent PIU association; and (3) whether the discrepancy (or gap) between parent and adolescent reports is associated with adolescent PIU, and whether this reflects a genuine non-linear effect or an asymmetry in which informant's report is more predictive. The conceptual model is presented in Fig. 1.

## 2 Method

### 2.1 Participants and procedure

Data were collected via Macromill, Inc., a commercial online research panel operating in Japan, using a specialized parent–adolescent dyad data collection course. Recruitment and eligibility screening proceeded in two stages prior to the main survey. First, panel members who had previously indicated living with a family member enrolled in high school were identified as a candidate pool. Second, this pool was sent a dedicated screening survey reconfirming household composition and internet use, and asking whether the parent could complete the questionnaires physically alongside their child without the adolescent viewing the parent's responses. Only respondents who indicated that this was possible, and who subsequently provided study-specific informed consent on behalf of both the parent and the adolescent (see below), were invited to the main survey. Within the main survey, the parent and adolescent completed their respective questionnaires sequentially on the same mobile device, with the parent responding first. This sequential, same-device procedure served two purposes: confirming parental consent in real time and verifying that the parent and adolescent belonged to the same family dyad. As in the parallel scale-development study using the same framework (Morita, in press), on-screen instructions asked the parent to step away after responding, and the parent–adolescent pairing was linked to the parent's authenticated household account; adherence to the step-away instruction could not be independently verified. A total of 842 dyads completed the main survey; as a panel-wide quality-control measure, the fastest-responding 18 (2.1%) were mandatorily excluded (likely removing low-effort or duplicated responses), yielding the final analytic sample of 824 dyads. The survey company aimed to recruit broadly across Japan's major regions; as is typical for such panel courses, exact screening-stage participation figures were not available to us, so we report the achieved sample (Table 1) and address generalizability implications in the Limitations. Participants received panel points (redeemable for cash or goods) for completing the survey. The sample consisted of 824 pairs of high school students (aged 15–20, mean 16.7 ± 0.8 years, Japanese) and their biological parents. Participant characteristics are summarized in Table 1. The predominance of male parent respondents (59.7%; see Table 1 for full characteristics) is atypical relative to most family survey research, in which mothers typically predominate as informants; we return to this point in the Discussion. Regarding age range, the large majority of adolescents were 15–18 years old (*n* = 820, 99.5%), consistent with typical Japanese high school enrollment; four (0.5%) were 19–20, attributable to grade retention or Japan's April-start academic year. A sensitivity analysis excluding these four cases yielded a virtually identical pattern (e.g., low-barriers discrepancy effect: β = 0.143 vs. 0.142), so all 824 dyads were retained. Adolescent sex was coded as male, female, or other/prefer not to answer (*n* = 5, 0.6%, entered as a continuous third category); a sensitivity analysis dummy-coding sex as male/female and excluding these five cases produced a materially identical pattern (β = 0.139), indicating the coding did not influence our conclusions. Inclusion criteria were: (1) parents and adolescents lived together, (2) they were biologically related, and (3) both reported using the internet. The survey was conducted between February 20 and March 2, 2026. The platform minimized missing data through system-level controls, and no missing values were identified in the final dataset. These procedures were consistent with those used in the scale development study (Morita, in press).

**Table 1:** Participant characteristics.

Variable	Category	*n*	%
Parent gender	Male	492	59.7
	Female	332	40.3
Parent age	30s	16	1.9
	40s	369	44.8
	50s	391	47.5
	60s and older	48	5.8
Child gender	Male	472	57.3
	Female	347	42.1
	Other (prefer not to answer)	5	0.6
Employment status	Full-time employee	554	67.2
	Stay-at-home parent	114	13.8
	Part-time, temporary, or other	150	18.2
	Unemployed	6	0.7
Education	Junior high school graduate	12	1.5
	High school graduate	185	22.5
	Junior college or vocational school graduate	203	24.6
	College or graduate school graduate	424	51.5

Participation was voluntary and anonymous. The parent provided informed consent for both their own and their adolescent's participation; the adolescent separately received an age-appropriate explanation and provided assent. The study protocol was approved by the authors' institutional ethics committee [masked for review] (Approval No. 2236).

## 3 Measures

### 3.1 Problematic internet use

Problematic internet use was assessed using the Diagnostic Questionnaire (DQ; Young, 1998), an 8-item dichotomous (“yes”/“no”) measure based on DSM-IV criteria for pathological gambling, with higher scores indicating greater PIU (Beard & Wolf, 2001, proposed a modification to the DQ's diagnostic criteria rather than establishing its validity; we cite this work only for that purpose). In the present sample, internal consistency (KR-20, appropriate for dichotomous items) was 0.812 for the parent version and 0.849 for the adolescent version. We retained the DQ, despite newer continuous-scale instruments (e.g., CIUS, PIUQ-SF), for continuity with the companion scale-development study (Morita, in press) and prior Japanese PIU research (e.g., Mihara et al., 2016); we acknowledge this as a limitation in the Discussion.

Separate DQ scores were computed for parents and adolescents.

### 3.2 Parent–child communication

Parent–child communication regarding internet use was assessed using the Parent–Child Internet Communication Scale (PCICS; Morita, in press), a measure with parallel parent-report and child-report versions. The parent version comprises three subscales: Dialogic parental involvement (5 items), parental acceptance (4 items), and low perceived barriers to consultation (3 items; total 12 items), with reliability coefficients of 0.84, 0.87, and 0.79, respectively. The child version comprises two subscales: parental acceptance (4 items) and low perceived barriers to consultation (4 items; total 8 items), with reliability coefficients of 0.88 and 0.72, respectively. The factor structure and psychometric properties of both versions have been confirmed through exploratory and confirmatory factor analyses. Because the parent and child versions are not fully parallel in item wording or number (e.g., three vs. four items for low perceived barriers to consultation; a dialogic-involvement subscale present only in the parent version), cross-informant comparability cannot be assumed a priori. This was addressed in the scale-development study: multi-group confirmatory factor analysis on a conceptually matched core item subset supported both configural and full metric invariance across versions (ΔCFI = −0.002, within the > −0.010 threshold; Chen, 2007; Morita, in press), providing empirical support for treating parental acceptance and low perceived barriers to consultation as measuring equivalent constructs across informants. Illustrative adolescent-version items include “My parents understand how I feel about the internet right now” (parental acceptance) and “I hesitate to discuss how I use the internet with my parents” (reverse-scored; low perceived barriers to consultation); full item sets for both versions appear in the companion scale-development study (Morita, in press).

The use of parallel reports reflects a multi-informant approach increasingly recommended in family research (De Los Reyes, 2013). Analyses focused on conceptually corresponding dimensions across parent and child reports—namely, parental acceptance and low perceived barriers to consultation. In the following analyses and tables, low perceived barriers to consultation is referred to as “Low barriers” for brevity.

### 3.3 Construction of mean and discrepancy variables

To examine both overall levels of communication and discrepancies between parent and adolescent perceptions, composite variables were constructed following a multi-informant framework (De Los Reyes, 2013).

First, parent and adolescent scores for each subscale were standardized (z-scores) to ensure comparability across informants.

Second, mean-level communication variables were computed as the average of standardized parent and adolescent scores:

Mean = (Z_parent + Z_child) / 2.

Third, discrepancy variables were computed as the difference between standardized parent and adolescent scores:

Gap = Z_parent − Z_child.

Positive values indicate that parents reported higher levels of communication than adolescents, whereas negative values indicate higher adolescent-reported levels.

### 3.4 Statistical analysis

We conducted zero-order correlations among study variables.

Hierarchical regressions were conducted using SPSS.

Step 1 entered child sex, child age, and parental PIU as control variables.

In Step 2, mean-level communication variables (parental acceptance and low perceived barriers to consultation) were entered to examine direct (protective) effects.

In Step 3, discrepancy variables (gap in parental acceptance and gap in low perceived barriers to consultation) were added to assess the effects of perceptual differences.

To test whether communication buffered the parent–adolescent PIU association, separate moderation analyses were conducted for each communication dimension, with interaction terms between standardized parental PIU and each mean-level communication variable (all variables standardized to reduce multicollinearity; Aiken & West, 1991).

Additional analyses including parent sex and parent age as covariates were conducted to assess the robustness of the findings.

Statistical significance was set at *p* < .05.

## 4 Results

Table 2 presents zero-order correlations. Parental PIU was positively correlated with adolescent PIU (*r* = 0.54, *p* < .001), and adolescent PIU showed significant negative correlations with all mean-level communication variables, consistent with the regression findings below.

**Table 2:** Correlation analysis between variables.

	DQ score (parent)	DQ score (child)	z-score of Parental acceptance (parent)	z-score of Low barriers (parent)	z-score of Parental acceptance (child)	z-score of Low barriers (child)	Parental acceptance (discrepancy)	Low barriers (discrepancy)	Mean of Parental acceptance
DQ score (parent)	1								
DQ score (child)	0.535***	1							
z-score of Parental acceptance (parent)	−0.058	−0.104**	1						
z-score of Low barriers (parent)	−0.253***	−0.169***	−0.057	1					
z-score of Parental acceptance (child)	−0.184***	−0.198***	0.556***	0.210***	1				
z-score of Low barriers (child)	−0.223***	−0.310***	0.116***	0.361***	0.161***	1			
Parental acceptance (discrepancy)	0.134***	0.100**	0.471***	−0.283***	−0.471***	−0.047	1		
Low barriers (discrepancy)	−0.027	0.125***	−0.153***	0.565***	0.043	−0.565***	−0.209***	1	
Mean of Parental acceptance	−0.137***	−0.171***	0.882***	0.086*	0.882***	0.157***	0	−0.063	1
Mean of Low barriers	−0.288***	−0.290***	0.036	0.825***	0.225***	0.825***	−0.201***	0	0.147***
M	1.60	1.96	0.00	−0.00	−0.00	−0.00	0.00	−0.00	−0.00
SD	2.19	2.49	1.00	1.00	1.00	1.00	0.94	1.13	0.88

Pearson's correlation coefficient, * *p* < .05, **p < .01, ***p < .001, “Low barriers” refers to *low perceived barriers to consultation*. M and SD for Mean of Low barriers (not shown as a column above) were − 0.00 and 0.82, respectively.

Hierarchical regressions examined the role of parent–child communication in adolescent PIU (Table 3); variance inflation factors ranged from 1.03 to 1.16, indicating no meaningful multicollinearity. Three sequential models addressed Research Questions 1 and 3: Model 1 entered the control variables (child sex, child age) together with parental PIU to establish the baseline intergenerational association; Model 2 added the mean-level communication variables to test the protective (direct) effect of communication; and Model 3 added the parent–adolescent discrepancy (gap) variables to test whether discrepancies, beyond mean levels, were associated with adolescent PIU. Research Question 2 (buffering) was tested separately, as reported below.

**Table 3:** Hierarchical regression predicting adolescent problematic internet use.

Variable	Model 1	Model 2	Model 3
Child sex	B = −0.33, SE = 0.14, 95% CI [−0.61, −0.05], β = −0.07*	B = −0.22, SE = 0.14, 95% CI [−0.50, 0.07], β = −0.04	B = −0.20, SE = 0.14, 95% CI [−0.48, 0.08], β = −0.04
Child age	B = −0.17, SE = 0.09, 95% CI [−0.35, 0.02], β = −0.05	B = −0.09, SE = 0.09, 95% CI [−0.28, 0.09], β = −0.03	B = −0.13, SE = 0.09, 95% CI [−0.31, 0.05], β = −0.04
Parent PIU	B = 0.61, SE = 0.03, 95% CI [0.54, 0.67], β = 0.53***	B = 0.55, SE = 0.03, 95% CI [0.49, 0.62], β = 0.49***	B = 0.56, SE = 0.03, 95% CI [0.49, 0.62], β = 0.49***
Parental acceptance (mean-level)		B = −0.22, SE = 0.08, 95% CI [−0.38, −0.05], β = −0.08**	B = −0.19, SE = 0.08, 95% CI [−0.36, −0.03], β = −0.07*
Low barriers (mean-level)		B = −0.40, SE = 0.09, 95% CI [−0.58, −0.21], β = −0.13***	B = −0.37, SE = 0.09, 95% CI [−0.56, −0.19], β = −0.12***
Parental acceptance (discrepancy)			B = 0.09, SE = 0.08, 95% CI [−0.06, 0.25], β = 0.04
Low barriers (discrepancy)			B = 0.31, SE = 0.07, 95% CI [0.19, 0.44], β = 0.14***

*R*^*2*^ = 0.30 / 0.32 / 0.34

*Adjusted R*^*2*^ = 0.30 / 0.31 / 0.33

*ΔR*^*2*^ = — / 0.02*** / 0.02***.

Note. PIU = problematic internet use, “Low barriers” refers to *low perceived barriers to consultation*. * *p* < .05, ** *p* < .01, *** *p* < .001.

In Model 1, parental problematic internet use was positively associated with adolescent problematic internet use (*β* = 0.53, *p* < .001), indicating a strong intergenerational association. Child sex was significantly associated with adolescent PIU (β = −0.07, *p* < .05), whereas child age was not significant.

In Model 2, mean-level communication variables were added. Both parental acceptance (*β* = −0.08, *p* < .05) and low perceived barriers to consultation (*β* = −0.13, *p* < .001) were significantly associated with lower adolescent PIU, explaining additional variance (*ΔR*^*2*^ = 0.02, *p* < .001), indicating a protective effect of communication.

**Fig. 2:**
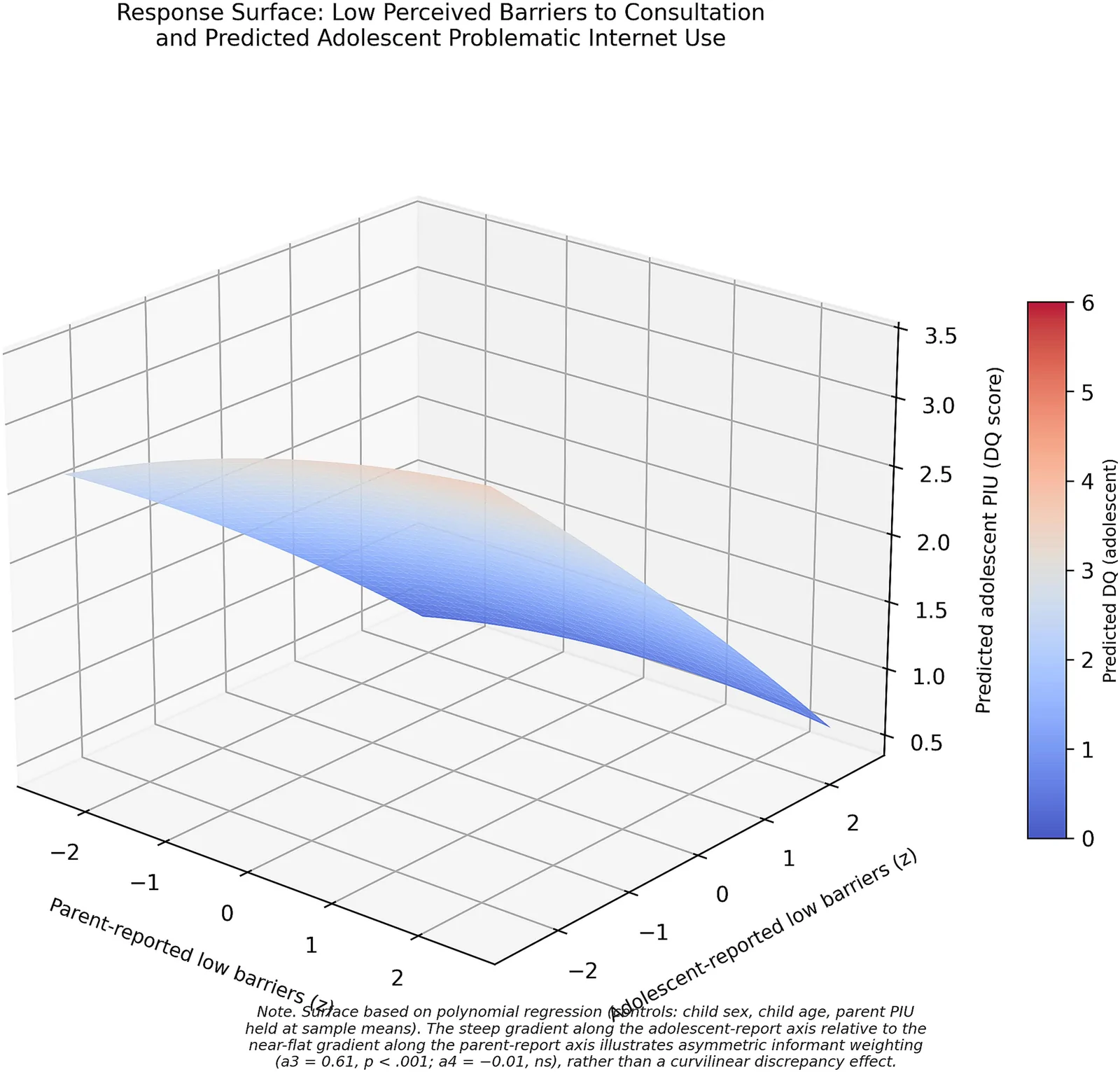
**Response surface for low perceived barriers to consultation and predicted adolescent problematic internet use.** The surface, derived from polynomial regression (Edwards & Parry, 1993), plots predicted adolescent DQ scores as a joint function of standardized parent- and adolescent-reported low barriers to consultation (control variables held at sample means). The steep gradient along the adolescent-report axis relative to the near-flat gradient along the parent-report axis illustrates that this association reflects asymmetric informant weighting (a3 = 0.61, *p* < .001; a4 = −0.01, ns) rather than a curvilinear discrepancy effect.

In Model 3, discrepancy variables were included. The discrepancy in low perceived barriers to consultation was significantly associated with higher adolescent PIU (*β* = 0.14, *p* < .001), whereas the discrepancy in parental acceptance was not significant (*β* = 0.04, *p* = .163). This association is illustrated in Fig. 2. This step accounted for an additional 2% of the variance (*ΔR*^*2*^ = 0.02, *p* < .001), indicating this term captured variance beyond the mean-level variables. Because difference scores are mathematically equivalent to a reparameterization of the individual reports (Edwards, 1994, Edwards, 2001), we examined this further using polynomial regression and response surface analysis (RSA; Edwards & Parry, 1993).

For low perceived barriers to consultation, the RSA surface parameter along the line of incongruence (a3, reflecting the difference in the slopes for parent and adolescent reports) was significant (a3 = 0.61, *SE* = 0.13, *p* < .001), whereas the curvature parameter along that line (a4) was not (a4 = −0.01, *ns*). This pattern indicates an asymmetric weighting of informants rather than a curvilinear discrepancy effect: adolescents' own reports of low barriers were strongly associated with adolescent problematic internet use (*β* = −0.51), whereas parents' reports were not (*β* = 0.10, *ns*). For parental acceptance, only the congruence (level) slope was significant (a1 = −0.26, *p* = .002), with no significant incongruence effect, consistent with a level-based rather than discrepancy-based effect.

A separate moderation analysis addressed Research Question 2, examining whether communication buffered the parent–adolescent PIU association (Table 4). Parental PIU (β = 0.52, p < .001) and parental acceptance (β = −0.09, *p* < .01) were significant predictors, but the interaction term was not (β = 0.00, *p* = .942; ΔR^2^ = 0.000), indicating no moderation. The interaction between parental PIU and low perceived barriers to consultation was also non-significant (β = −0.04, *p* = .209), indicating the absence of buffering was consistent across both dimensions.

**Table 4:** Moderation analysis of parent–child communication.

Variable	*Model 1: Parental acceptance × Parent PIU*	*Model 2: Low barriers × Parent PIU*
Child sex	B = −0.28, SE = 0.15, 95% CI [−0.56, 0.01], β = −0.06	B = −0.27, SE = 0.14, 95% CI [−0.55, 0.02], β = −0.05
Child age	B = −0.14, SE = 0.09, 95% CI [−0.32, 0.05], β = −0.04	B = −0.11, SE = 0.09, 95% CI [−0.29, 0.07], β = −0.03
Parent PIU	B = 1.30, SE = 0.08, 95% CI [1.15, 1.45], β = 0.52***	B = 1.20, SE = 0.08, 95% CI [1.05, 1.36], β = 0.48***
Communication (mean-level)	B = −0.25, SE = 0.09, 95% CI [−0.42, −0.08], β = −0.09**	B = −0.41, SE = 0.09, 95% CI [−0.59, −0.23], β = −0.14***
Parent PIU × Communication (interaction)	B = 0.01, SE = 0.09, 95% CI [−0.17, 0.19], β = 0.00	B = −0.10, SE = 0.08, 95% CI [−0.26, 0.06], β = −0.04

*R*^*2*^ = 0.31

*Adjusted R*^*2*^ = 0.30

*ΔR*^*2*^ = 0.000, not significant.

Note. PIU = problematic internet use. “Low barriers” refers to *low perceived barriers to consultation*. * *p* < .05, ** *p* < .01, *** *p* < .001.

All variables were standardized prior to computing interaction terms.

Additional analyses including parent sex and age as covariates yielded essentially the same pattern, confirming robustness.

Because the DQ is a count measure with substantial zero-inflation (47.8% of adolescents scored 0), we conducted sensitivity analyses re-estimating the Step 3 model using Poisson regression, negative binomial regression (NB2; dispersion α = 1.10, confirming overdispersion), and heteroscedasticity-robust (HC3) OLS. Results for the mean-level communication and low-barriers discrepancy effects were consistent across specifications; the parental-acceptance discrepancy reached significance only under Poisson regression (*p* = .011, vs. *p* = .26–0.34 elsewhere), so we do not interpret it as robust.

## 5 Discussion

First, parental PIU was strongly associated with adolescent PIU, supporting intergenerational processes—consistent with prior research on modeling, shared environments, and family norms.

Second, higher parent–child communication was associated with lower PIU, indicating a protective effect, in line with prior parental mediation research emphasizing supportive, communicative parenting.

Third, and a key contribution of this study, communication did not moderate the parent–adolescent PIU association for either dimension examined, indicating that it does not function as the buffering mechanism often assumed in the literature. Instead, parental influence and communication appear to operate through partially independent pathways—communication may reduce overall risk without attenuating intergenerational transmission.

This lack of buffering may reflect several mechanisms. First, the strong intergenerational association (*β* = 0.53, *p* < .001) suggests social modeling of parental internet use may be too pervasive to be neutralized by verbal communication alone: per Social Learning Theory (Bandura, 1977), adolescents internalize observed behaviors more deeply than verbal instructions, and parental smartphone preoccupation (“phubbing”) predicts adolescent PIU regardless of relationship quality (Ma et al., 2024). Second, the direct pathway may reflect shared genetic or temperamental vulnerabilities (e.g., low self-control, impulsivity) linked to PIU (Kim et al., 2017; Li et al., 2016; Pino et al., 2024).

Fourth, addressing Research Question 3, low perceived barriers to consultation showed no distinct discrepancy-based risk pathway: RSA indicated this association was primarily driven by adolescents' own perceptions, largely independent of parents'. Discrepancies in parental acceptance showed no comparable association once level effects were accounted for.

Parental acceptance reflects a general relational climate—whether adolescents feel understood and supported in everyday interactions—whereas low perceived barriers to consultation relates more directly to help-seeking in situations involving difficulties.

These findings suggest that what matters most is not whether parent and adolescent perceptions of accessibility align, but the adolescent's own subjective sense of whether they can approach their parents when facing difficulties.

This interpretation is consistent with multi-informant research suggesting adolescent self-report is often the more proximal predictor of adolescent-reported outcomes, particularly for constructs tied to adolescents' own subjective experience (De Los Reyes, 2013). Our findings extend this by showing that the apparent “discrepancy effect” for help-seeking barriers is better characterized as reflecting the primacy of adolescents' own perceptions rather than a non-linear mismatch effect.

In a robustness model adding parent sex and parent age as covariates to Model 3 (reported in-text), parent sex showed a significant independent association with adolescent PIU (β = 0.12, *p* < .001), and parent age a smaller but significant association (β = 0.06, *p* = .040); the main communication and discrepancy findings remained robust after controlling for both, suggesting the core associations reflect general family communication processes rather than differences specific to mothers or fathers, though the parent sex association itself warrants further attention (see Limitations).

### 5.1 Practical implications

From a practical perspective, findings suggest interventions should attend not only to the frequency or quality of parent–child communication but also to adolescents' own subjective sense of accessibility in help-seeking contexts, since adolescents' reports of low barriers were associated with PIU largely independent of parents' reports. Given the small effect sizes and cross-sectional design, we offer this cautiously: family-based programs might consider incorporating opportunities for adolescents to voice their sense of parental accessibility, rather than assuming parents' own beliefs suffice—a direction warranting further investigation rather than a validated intervention target.

Two considerations temper these implications. First, given the modest effect sizes, we are mindful of the risk of overpathologizing everyday adolescent internet use, since many adolescents flagged by screening instruments do not show functional impairment consistent with a clinical disorder (Nogueira-López et al., 2023). Second, translating such findings into effective prevention programs has proven difficult in practice, with recent reviews documenting substantial heterogeneity in program effectiveness (Gómez et al., 2026; Villanueva-Blasco et al., 2025); we therefore present our findings as informative for theory and future research rather than a direct intervention blueprint.

### 5.2 Limitations

Despite these insights, several limitations should be considered. First, the cross-sectional design precludes definitive conclusions about causal direction; longitudinal studies are needed to clarify developmental trajectories. Second, self-report data may be subject to social desirability or recall bias. Third, the commercial online survey panel may limit generalizability. Beyond the formal inclusion criteria, at least two selection mechanisms plausibly inflated the observed parent–adolescent correlation: parents registered with a points-based panel are likely relatively heavy internet users themselves, and parents already concerned about their child's internet use may have been more motivated to opt into a survey framed around adolescent internet use—a concern-based self-selection process. These dynamics should be considered alongside Japan's rapidly increasing smartphone use since the COVID-19 pandemic. More broadly, because participants were drawn from an opt-in panel via a specialized parent–adolescent dyad course, the source population is not well defined, and exact invitation/participation figures were unavailable to us (see Participants and Procedure); geographic diversification does not offset this more fundamental self-selection. Caution is therefore warranted in generalizing to more diverse or clinical populations. Relatedly, because this study involved minors, the parent and adolescent completed questionnaires sequentially on the same device, with the parent's response confirming consent (see Participants and Procedure); we cannot rule out that this allowed one respondent's answers to influence the other's. Several considerations partly mitigate this: the fastest-responding 18 respondents (2.1%) were mandatorily excluded; gap scores showed substantial variability rather than clustering near zero; and the parent–adolescent correlation for an unrelated construct (psychological distress, K6; *r* = 0.62) was comparable to or greater than the PIU correlation, suggesting elevated cross-informant agreement was general rather than specific to PIU. Independent verification (e.g., separate devices) was not possible given the consent procedure required for minors. Male respondents also predominated among parent informants (59.7%), atypical for family communication research; although parent sex was statistically controlled, findings may not generalize equally to families where mothers are the primary informant. Fourth, we retained the DQ (Young, 1998), developed for adolescents, for both parents and adolescents; newer continuous-scale instruments (e.g., CIUS, PIUQ-SF) may offer superior psychometrics for adults, and future work should consider replication with such measures. Fifth, this study used a Japanese sample; attitudes toward help-seeking and parent–child disclosure vary cross-culturally (Lam & Zane, 2004; Nucci et al., 2014); discrepancy effects may differ in more individualistic societies, and future cross-cultural studies are needed to test whether these pathways replicate. Finally, we examined only two of the three PCICS dimensions, selected because parallel parent- and adolescent-report versions existed; dialogic parental involvement, assessed only from the parent's perspective, was not included, and future research incorporating a broader range of communication domains would provide a more complete picture.

## 6 Conclusion

The present study provides a refined understanding of parent–child communication in adolescent problematic internet use. While communication was associated with lower PIU, it did not buffer the parent–adolescent association, suggesting it functions as an independent protective factor rather than a mechanism that attenuates intergenerational risk.

Importantly, adolescents' own reports of **low perceived barriers to consultation**, rather than parent–adolescent discrepancies per se, emerged as the primary correlate of risk, whereas **parental acceptance** operated through overall level rather than discrepancy. This pattern underscores the importance of adolescents' subjective experience of accessibility in help-seeking contexts, though the small effect sizes and cross-sectional design warrant caution in drawing intervention implications.

These results extend existing parental mediation frameworks by emphasizing the importance of adolescents' own perceptions and domain-specific communication processes within families.

## CRediT authorship contribution statement

**Nobuaki Morita:** Writing – review & editing, Writing – original draft, Visualization, Validation, Resources, Project administration, Methodology, Investigation, Funding acquisition, Formal analysis, Data curation, Conceptualization.

## Consent for publication

Not applicable.

## Ethics approval and consent to participate

The study was conducted with the approval of the first author's institutional Ethics Committee (Approval No. 2236). Written informed consent was obtained from parents, and adolescents provided assent.

## Funding

This work was supported by the 10.13039/501100003478Ministry of Health, Labour and Welfare, Japan (FY2025 Research Project on Addiction; Grant Number: 202605).

## Declaration of competing interest

The author declares that there are no known competing financial interests or personal relationships that could have appeared to influence the work reported in this paper.

## Data Availability

Data will be made available on request.
